# Photoacoustic signal enhancement in dual-contrast gastrin-releasing peptide receptor-targeted nanobubbles

**DOI:** 10.3389/fbioe.2023.1102651

**Published:** 2023-01-17

**Authors:** Shensheng Zhao, Leanne Lee, Yang Zhao, Nu-Chu Liang, Yun-Sheng Chen

**Affiliations:** ^1^ Department of Electrical and Computer Engineering, University of Illinois Urbana-Champaign, Urbana, IL, United States; ^2^ Beckman Institute for Advanced Science and Technology, University of Illinois Urbana-Champaign, Urbana, IL, United States.; ^3^ Department of Bioengineering, University of Illinois Urbana-Champaign, Urbana, IL, United States; ^4^ Carl R. Woese Institute for Genomic Biology, University of Illinois Urbana-Champaign, Urbana, IL, United States; ^5^ Department of Psychology, University of Illinois Urbana-Champaign, Urbana, IL, United States; ^6^ Department of Biomedical and Translational Sciences, University of Illinois Urbana-Champaign, Urbana, IL, United States

**Keywords:** cancer diagnosis, photoacoustic, ultrasound, molecular imaging, multimodal imaging, nanobubbles, GRPR, ICG

## Abstract

Translatable imaging agents are a crucial element of successful molecular imaging. Photoacoustic molecular imaging relies on optical absorbing materials to generate a sufficient signal. However, few materials approved for human use can generate adequate photoacoustic responses. Here we report a new nanoengineering approach to further improve photoacoustic response from biocompatible materials. Our study shows that when optical absorbers are incorporated into the shell of a gaseous nanobubble, their photoacoustic signal can be significantly enhanced compared to the original form. As an example, we constructed nanobubbles using biocompatible indocyanine green (ICG) and biodegradable poly(lactic-co-glycolic acid) (PLGA). We demonstrated that these ICG nanobubbles generate a strong ultrasound signal and almost four-fold photoacoustic signal compared to the same concentration of ICG solution; our theoretical calculations corroborate this effect and elucidate the origin of the photoacoustic enhancement. To demonstrate their molecular imaging performance, we conjugated gastrin-releasing peptide receptor (GRPR) targeting ligands with the ICG nanobubbles. Our dual photoacoustic/ultrasound molecular imaging shows a more than three-fold enhancement in targeting specificity of the GRPR-targeted ICG nanobubbles, compared to untargeted nanobubbles or prostate cancer cells not expressing GRPR, in a prostate cancer xenograft mouse model *in vivo*.

## 1 Introduction

Photoacoustic imaging is an emerging diagnostic imaging in clinics for health conditions such as breast cancers, skin cancers, vascular dysfunctions, and wounds (Mallidi et al., 2011; Attia et al., 2019; Manohar and Gambhir, 2020). When incorporated with disease-targeting agents (Weber et al., 2016; Zhao et al., 2022), photoacoustic molecular imaging enables the *in vivo* visualization and characterization of biological processes from various animal models (Wilson et al., 2017; Chen et al., 2019; Qiu et al., 2020; Xie et al., 2020; Nguyen et al., 2021; Zhou et al., 2022). It has become a necessary imaging tool to study cancer biology, neuroscience, and immunology in preclinical research. Photoacoustic imaging detects the acoustic signal from optical absorption-induced photothermal expansion (Beard, 2011; Wang and Yao, 2016). An ultrasound imaging system can collect the signal transmitted from imaging targets to reconstruct a photoacoustic image. Because photoacoustic and ultrasound imaging shares the same imaging apparatus, both techniques can seamlessly integrate into one system. Dual imaging systems are popular because of the versatile photoacoustic imaging agents and the rich tissue information of ultrasound images (Song et al., 2009; Luke et al., 2013; Kim et al., 2020; Jeng et al., 2021; Karlas et al., 2021; Robin et al., 2021; Park et al., 2022). Ultrasound imaging alone is capable of molecular imaging as well (Abou-Elkacem et al., 2015; van Rooij et al., 2015; Kosareva et al., 2020). Thus, dual-photoacoustic/ultrasound molecular imaging, which is expected to increase diagnostic sensitivity, can be achieved using dual-modality imaging agents.

Molecular imaging agents that can be safely used with humans are critical for a successful clinical translation. The recent development of clinical photoacoustic molecular imaging largely focuses on constructing imaging agents from materials with well-studied safety profiles to minimize the risk of unexpected toxicity. However, these materials may not generate sufficient photoacoustic response due to their limited optical absorption. The low photoacoustic signal raises the minimum-detectable molecular agent threshold that hinders clinical applications. Recent studies have shown that it is possible to further improve photoacoustic characteristics through engineering the coating (Chen et al., 2011; Moon et al., 2015), physical properties (Chen et al., 2019), and aggregation of nanoparticles (Chen et al., 2017; Park et al., 2018). The nanoengineering approach paves a new strategy to enhance the photoacoustic signal of imaging agents made of biocompatible materials.

Dual ultrasound/photoacoustic imaging agents are typically composed of optical absorbing materials and echogenic micro- and nanoparticles. Within the dual imaging agents, dye-doped micro- and nanobubbles are considered highly translatable dual agents because their compositions are FDA-approved materials such as indocyanine green (ICG) dye, lipid or biodegradable poly(lactic-co-glycolic acid) (PLGA) (Xu et al., 2009; Wang et al., 2016; Wang et al., 2020; Yang et al., 2020; Yin et al., 2020). The ICG dyes of a nanobubble usually are distributed within a confined and thin space on the shell. The photoacoustic pressure generated from the ICG absorption would interact with a highly compressible elastic gas core; thus, the photoacoustic signal generated from ICG nanobubbles could be very different from free ICG solutions. This nanoscale photoacoustic effect could add an important degree of freedom in developing a highly efficient photoacoustic imaging agent.

Here, we investigate the effect of the gas bubbles on the photoacoustic characteristics. In this study, we chose PLGA as a constitutional material to construct ICG-nanobubbles. PLGA is a FDA-approved copolymer for use in therapeutic devices. Due to its excellent biocompatibility, ICG-PLGA nanoparticles for photoacoustic imaging applications are well-documented (Kohl et al., 2011; Wang et al., 2014; Liu et al., 2018; Changalvaie et al., 2019; Shen et al., 2019; Xiong et al., 2019). The main advantage of encapsulating ICG in PLGA for photoacoustic imaging is that the PLGA matrix can thermally stabilize the ICG dye, which is critical for photoacoustic applications because high-intensity laser pulses are used in imaging. While ICG-PLGA nanobubble of photoacoustic phantom imaging was reported (Xu et al., 2009), its *in vivo* application in photoacoustic molecular imaging has not been explored, and neither did the effect of PLGA gas bubbles on the photoacoustic signal. In this study, we created ICG-PLGA nanobubbles and showed that gas bubbles enhance the photoacoustic signal of ICG dyes up to four-fold. Our study suggests the oscillation of gas bubbles is an important factor contributed to the photoacoustic signal enhancement. With the ICG-PLGA nanobubbles, we demonstrated dual *in vivo* photoacoustic/ultrasound molecular imaging of gastrin-releasing peptide receptor (GRPR) expression in a mouse xenograft prostate tumor model. The results show targeting specificity enhanced by more than three-fold, compared to untargeted nanobubbles or prostate cancer cells not expressing GRPR.

## 2 Materials and methods

### 2.1 Materials

All chemicals in this study were used as received: poly(lactide-co-glycolide) (PLGA, Mw 54,000–69,000, PolySciTech), poly(lactide-co-glycolide)-b-poly(ethylene glycol) (PLGA-PEG, PolySciTech), poly(lactide-co-glycolide)-b-poly(ethylene glycol)-maleimide (PLGA-PEG-Mal, PolySciTech), poly(vinyl alcohol) (PVA, average Mw 13,000–23,000, Sigma-Aldrich), dichloromethane (Sigma-Aldrich), indocyanine green (ICG, Sigma-Aldrich) Growth factor reduced Matrigel (Corning), Phosphate-buffered saline (PBS, Thermo Fisher Scientific), Presto Blue (Invitrogen), anti-GRPR antibody (AbCam, ∼48 kDa). Selenocystamine (Sigma-Aldrich), Dithiothreitol (Sigma-Aldrich).

### 2.2 Synthesis of ICG nanobubbles

Briefly, 100 mg of ICG was dissolved in 1 mL of PVA solution (1 w/v%). Two hundred microliter of the ICG-PVA solution was added in drops to 10 mL of PLGA/dichloromethane (0.25 w/v %) in an ice-water bath under sonication to form the first emulsion. Here, the PLGA polymer contains the mixture of 80 wt% of PLGA, 10 wt% of poly(lactide-co-glycolide)-b-poly(ethylene glycol) (PLGA-PEG), and 10 wt% of poly(lactide-co-glycolide)-b-poly(ethylene glycol)-maleimide (PLGA-PEG-MAL) copolymer. Typically, for nanobubble around 250 nm in diameter, the sonicator (Branson Sonifer 450) was set in a pulse width of 5 s on and 5 s off period, the sonication power was 20% of total power, and the sonication period was 5 min in total. The first emulsion was transferred and added dropwise to 10 mL of PVA solution (1 w/v %) under sonication (5 s on-off in 20% power for 5 min) in an ice-water bath to form second emulsions. The second emulsion was poured slowly to 20 mL of isopropanol (5% v/v) in DI water solution and stirred for more than 2 h with magnetic stirring. The ICG nanoparticles were centrifuged at 1,500 rpm for 20 min, re-suspend with water and washed twice with a centrifuge. The ICG nanoparticles were then lyophilized for 48 h to form ICG nanobubbles.

### 2.3 Characterization of ICG nanobubbles

The optical absorption of the ICG nanobubbles was characterized using ultraviolet-to-visible (UV-Vis) extinction spectroscopy. Extinction spectra were collected from a 0.1 mL nanobubble suspension in a Microplate Reader (BioTek, Synergy) at room temperature. The average size of the ICG nanobubbles was measured with a dynamic light scattering system (DLS, Zetasizer Nano ZS, Malvern) at room temperature, and the size distribution of nanobubbles was determined by the polydispersity index (PDI). The concentrations of the nanobubbles were measured by nanoparticle tracking analysis (Nanosight NS300, Malvern) at 25°C. The morphology of the nanobubbles was assessed by environmental scanning electro-microscopy (Zeiss Sigma FESEM).

The concentration of ICG in the ICG PLGA nanobubble solution is calculated by subtracting the initial ICG mass by the mass of the free ICG in the solution. Specifically, the nanobubbles and supernatant in an as-synthesized ICG PLGA nanobubble solution were separated by a centrifuged filter unit (Millipore, 100K MWCO). The optical density of the supernatant was measured by UV-Vis. The optical density at 780 nm is used to calculate the mass of the free ICG dye in the supernatant with a pre-calibrated standard curve, indicating the optical absorption of the ICG solution as a function of concentration. The mass of ICG in PLGA nanobubbles solution was then calculated by subtracting the mass of initially added ICG by the mass of free ICG dyes in the supernatant. The concentration (μg/mL) is the ratio between the ICG mass and the volume of the solution.

### 2.4 Characterization of photoacoustic and ultrasound signal in ICG nanobubbles

To investigate the photoacoustic/ultrasound performance of ICG nanobubbles, we prepared ICG nanobubble/normal saline (0.9 w/v % sodium chloride) solution for each size of ICG nanobubbles, and matched their optical density (OD) across different sizes. Due to the difficulty in precisely control, we also prepared OD-matched ICG/normal saline solution as a control. The optical density of all solutions was matched to be the same (OD = 3.0 in 1 cm optical path). For visualizing the photoacoustic and ultrasound signal, an agarose tissue-mimicking phantom with four square inclusions containing gelatin mixed with ICG nanobubbles of different sizes or ICG solution. As shown in Figure 2, a 7.5 mm thick slab was made of 4 wt% of agarose mixed with 0.2 wt% of silica ultrasound scatterers (40 µm in diameter). Inside the slab, four square plastic spacers were placed to create four inclusions aligned horizontally 10 mm from the top surface of the phantom. The molds were filled with 400 µL of a one-to-one (vol:vol) mixture of a 10 wt% aqueous gelatin solution at 40°C–50°C and an aqueous solution of ICG nanobubbles of a particular size.

Prior to imaging, the phantom was stored in the fridge at 4°C. The programable ultrasound micro-imaging system was used to capture both ultrasound and photoacoustic signals. A 21 MHz array ultrasound transducer (MS250, VisualSonics, Inc.) was mounted on a one-dimensional positioning stage. The position of the transducer was adjusted so that the inclusion was in the focal region of the ultrasound transducer. The phantom was placed in a water cuvette with an optical window on one side. A laser beam (5–7 ns pulse duration, 10 Hz repetition rate) generated from a wavelength-tunable OPO laser system pumped by a pulsed Nd-YAG laser uniformly irradiated the phantom with inclusions through the optical window. The acquired ultrasound and photoacoustic images were captured in real-time and then processed offline. An average laser fluence of 5.5 mJ/cm^2^ was used for the imaging to prevent damaging the ICG and an average laser fluence of 10 mJ/cm^2^ was used for testing photoacoustic signal stability. The scanning area was 13.9 mm (width) × 14.9 mm (depth) × 27.6 mm (length) with a step size of 95 μm. The lateral and axial direction of the transducer is aligned with the cross-section of the phantom, and the scanning direction (the elevational direction of a transducer) is perpendicular to the phantom cross-section.

### 2.5 Photoacoustic signal calculation

We calculated the photoacoustic pressure of dye-coated nanobubbles. In the simulation, the nanobubbles are embedded in a homogenous, incompressible fluid and only pulsate in the radial direction. A Gaussian laser pulse with a full-width-at-half-maximum (FWHM) of 5 ns and a fluence of 20 mJ/cm^2^ is used to create ICG photoacoustic signal. In addition, we assume the light fluence is uniform, and there is no thermal energy diffusion from the particles to the surrounding liquid.

For the dye-coated nanobubble with a radius of R , we can use the Rayleigh’s model to solve the photoacoustic pressure distribution over time (Firouzi et al., 2013; Dixon et al., 2015). Assuming bubbles are in a spherical symmetry and Newtonian liquid, we can use the Navier-Stokes equation for motion(Leighton, 1994):

$$\rho_{L}\left(\frac{\partial u}{\partial t}+u\frac{\partial u}{\partial r}\right)=-\frac{\partial p}{\partial r}+\mu_{L}\left[\frac{1}{r^{2}}\frac{\partial }{\partial r}\left(r^{2}\frac{\partial u}{\partial r}\right)-\frac{2u}{r^{2}}\right],$$ (1)

where $\rho_{L}$ is the density of liquid, $u\left(r,t\right)$ is radial outward velocity, $p\left(r,t\right)$ is the pressure distribution and $\mu_{L}$ is the viscosity coefficient. Since the liquid is incompressible, the velocity u at a point r is $u\;\left(r,t\right)=\frac{\dot{R}R^{2}}{r^{2}}$ , then we have:

$$\rho_{L}\left(\frac{\ddot{R}R^{2}+2{\dot{R}}^{2}}{r^{2}}-\frac{{\dot{R}}^{2}R^{4}}{r^{5}}\right)=-\frac{\partial p}{\partial r}\;.$$ (2)

Integrating Equation 2 from r to ∞ , we obtain:

$$\rho_{L}\left(\frac{R}{r}\left(R\ddot{R}+2{\dot{R}}^{2}\right)-{\frac{1}{2}\left(\frac{R}{r}\right)}^{4}{\dot{R}}^{2}\right)=p\left(r,t\right)-P_{0},$$ (3)

where $P_{0}$ is the pressure at the location r→∞ and $p\left(r,t\right)$ is radiated pressure from the bubble oscillation. When r=R , we have:

$$\rho_{L}\left(R\ddot{R}+\frac{3}{2}{\dot{R}}^{2}\right)=p\left(R,t\right)-P_{0}.$$ (4)

At the position r=R , the dye-coated nanobubble has the boundary condition:

$$p_{PA}={\sigma_{rr}+p}_{G}-\frac{2\gamma }{R},$$ (5)

where $p_{PA}$ is the initial photoacoustic pressure,

$$\sigma_{rr}=-p\left(R,t\right)+2\mu_{L}\frac{\partial u}{\partial r}\left(R,t\right),$$ (6)

is the radially outward normal stress and $p_{G}$ is the gas pressure inside the bubble. γ is the surface tension. Since there is no heat diffusion into the gas-filled region,

$$p_{G}=p_{G0}{\left(\frac{R_{0}}{R}\right)}^{3\kappa }\;,$$ (7)

where $R_{0}$ is the initial size of the bubble and κ is the polytropic index. $p_{G0}$ is the initial gas pressure and $p_{G0}=P_{0}+\frac{2\gamma }{R_{0}}$ .

We can replace radial outward velocity u in (6) using $u=\frac{\dot{R}R^{2}}{r^{2}}$ and link (5)–(7) with $p_{G0}=P_{0}+\frac{2\gamma }{R_{0}}$ , then we have:

$$p\left(R,t\right)=\left(P_{0}+\frac{2\gamma }{R_{0}}\right){\left(\frac{R_{0}}{R}\right)}^{3\kappa }-\frac{2\gamma }{R}-4\mu_{L}\frac{\dot{R}}{R}-p_{PA}\;.$$ (8)

Using (8) to replace $p\left(R,t\right)$ in (4),

$$R\ddot{R}+\frac{3}{2}{\dot{R}}^{2}=\frac{1}{\rho_{L}}\left\{\left(P_{0}+\frac{2\gamma }{R_{0}}\right){\left(\frac{R_{0}}{R}\right)}^{3\kappa }-\frac{2\gamma }{R}-P_{0}{-4\mu_{L}\left(\frac{\dot{R}}{R}\right)-p}_{PA}\right\}.$$ (9)

Under thermal and stress confinement, the initial photoacoustic pressure is(Cox and Beard, 2005)

$$p_{PA}=\Gamma \mu_{a}\Phi \left(t\right),$$ (10)

where Γ is Grueneisen coefficient, $\mu_{a}$ is the absorption coefficient of the dye and $\Phi \left(t\right)$ is the laser fluence. In the simulation, we consider a pulsed laser source, which has the Gaussian pulse profile. The laser fluence is given as(Cox and Beard, 2005; Firouzi et al., 2013)

$$\Phi \left(t\right)=\Phi_{0}\sqrt{\pi }\exp\left({-\left(\frac{t-t_{0}}{t_{p}}\right)}^{2}\right),$$ (11)

where $t_{0}$ is the time delay and $t_{p}$ is the pulse width.Using (10) and (11) to replace $p_{PA}$ in (9), $R\ddot{R}+3/2{\dot{R}}^{2}$

$$=\frac{1}{\rho_{L}}\left\{\left(P_{0}+\frac{2\gamma }{R_{0}}\right){\left(\frac{R_{0}}{R}\right)}^{3\kappa }-\frac{2\gamma }{R}-P_{0}-4\mu_{L}\left(\frac{\dot{R}}{R}\right)-\Gamma \mu_{a}\Phi_{0}\sqrt{\pi }\exp\left({-\left(\frac{t-t_{0}}{t_{p}}\right)}^{2}\right)\right\}.$$ (12)

We used MATLAB solver function *ode45* to solve (12). Once the motion of the bubble wall ( R ) is determined, the photoacoustic pressure distribution of a dye-coated nanobubble can be estimated from (3). The values of the simulation parameters are shown in Supplementary Table S1.

### 2.6 GRPR antibody and ICG nanobubble conjugation

Anti-GRPR antibody and ICG nanobubble conjugation was achieved through a Michael addition reaction between the maleimide groups on the PLGA and the thiol groups reduced from the disulfide bonds of Anti-GRPR antibody. Specifically, the 800 μL antibody in EDTA-PBS solution (150 μg of antibodies, and 1 mM of EDTA) was mixed with selenocystamine solution (100 μL, 5 mM) and aqueous dithiothreitol (DTT) solution (100 μL, 10 mM) at room temperature for 5 min in the dark. Spin column (Bio-Rad spin 6 column) was used to separate DTT and diabodies. The reduced anti-GRPR antibody solution was then mixed with ICG-nanobubble (2 mg in 1 mL of PBS) solution for 30 min at room temperature. The 2-mercaptoethanesulfonate (MESNA) (15 μL, 0.1 M) was then added to quench the reaction for 1 min. Anti-GRPR-targeted nanobubbles were separated using a centrifugal filter unit (100 kDa, 200 g for 30 min twice at 4^o^C).

### 2.7 Cell culture

Human prostate cancer cell lines, PC3-GFP, and DU145-GFP cells were purchased from American Type Tissue Collection (ATCC) and cultured on collagen-coated flasks (BD Biosciences) in RPMI 1640 supplemented with 10% heat-inactivated fetal bovine serum per the manufacturer’s recommendation. The cultures were maintained in a humidified incubator with 5% CO_2_/95% air at 37°C. The cell lines were tested for mycoplasma contamination upon received, after thawing, and monthly during culture using MycoAlert Mycoplasma Detection Kit (Lonza).

### 2.8 Cell viability test

PC3 cells were plated in triplicate for each ICG nanobubble concentration (100 μL, four concentrations 1 × 10^9^ nanobubbles/mL, 1 × 10^10^ nanobubbles/mL, 1 × 10^11^ nanobubbles/mL, 3×10^12^ nanobubbles/mL, and a control 0.9 wt% saline) at a density of 1 × 10^4^ cells per well in a 96-well plate. Cells were allowed to attach for 24 h; then, the wells were washed with PBS. The 100 µL of the mixture of ICG nanobubbles in the medium was added to the wells and incubated in a cell incubator for 24 h. Nanobubbles and medium were then removed from the wells and replaced with 100 μL of medium and 10 μL of Presto Blue. The plate was incubated at 37 °C for 30 min before fluorometer reading at 590 nm (Excitation 560 nm, Synergy 4, BioTek).

### 2.9 Animal studies

All animal experiments were performed in compliance with the Institutional Animal Care and Use Committee established by the University of Illinois Urbana Champaign under the protocol IACUC- 19124. Healthy male nu/nu mice (Jackson Laboratory) at age 6 weeks were used in this study. Prostate cancers (PC3 and DU145 cancer cell lines) in the mouse models were developed by subcutaneously injecting 100 µL of 5×10^6^ prostate cancer cells mixed with a 1:1 volume ratio of growth factor reduced Matrigel (Corning) into the right flank of each mouse. The tumors were allowed to grow to about 1 cm^3^ before imaging. Mice were anesthetized with 2% isoflurane at 2 L/min^−1^ of oxygen flow. 100 µL of nanoparticles/phosphate-buffered saline solution (ICG nanobubbles in PBS, 1 × 10^11^ nanobubbles/mL) were injected into the mice through the tail vein.

### 2.10 *In vivo* photoacoustic/ultrasound imaging setup

For *in vivo* photoacoustic/ultrasound imaging, we used nanosecond laser pulses at 780 nm (Nd:YAG pumped wavelength tunable nanosecond OPO pulsed laser) with a fluence of 20 mJ/cm^2^, a pulse width of 5 ns, and the photoacoustic imaging transducer (MS250) with a center frequency of 21 MHz. A volume of 23 mm × 19 mm × 16 mm was mechanically scanned with a step size of 63 μm along the elevational direction. Given the imaging parameters, each 3D imaging scan thus required 252 frames.

### 2.11 Quantification of bio-distribution of ICG nanobubbles

We used *in vivo* fluorescence imaging to confirm the uptake of ICG nanobubbles in tumors. IVIS spectrum imaging system (PerkinElmer) with an excitation filter centered at 745 nm and an emission filter centered at 840 nm with 60 s of exposure (F/stop of 2, medium binning) was used to record the fluorescence images. We also quantified the ICG nanobubble distribution in the tissue of the main organs with the same fluorescence imaging. For the bio-distribution, mice were sacrificed 24 h post-injection of the nanobubbles and imaged by the IVIS system. We quantitatively analyzed the images using Living Image 4.5 software using the radiant as the read-out.

### 2.12 Data analyses, statistics, and reproducibility

We used MATLAB to process the images acquired with the ultrasound/photoacoustic imaging system. The ultrasound images are shown in dB scale and the photoacoustic images are in linear scale. Data plot, average, and standard deviation were computed in Origin pro-2019.

For the bio-distribution of the ICG nanobubbles in the *in vivo* studies, we summed the fluorescence intensities within the region of interest. The region of interest was identified by the footprint of each organ from the photographic images. We then normalized the summation with the footprint to obtain the mean and standard deviation. In this research, we calculated the two-tailed *p*-value using an unpaired student t-test to determine the significance. We considered our data to be statistically significant with *p* < 0.05.

The environmental-electron microscopy and the DLS size measurements were repeated three times independently with similar results; the optical fluorescence imaging was repeated 3 times independently with similar results.

## 3 Results

### 3.1 Characteristics of ICG nanobubbles

**FIGURE 1:**
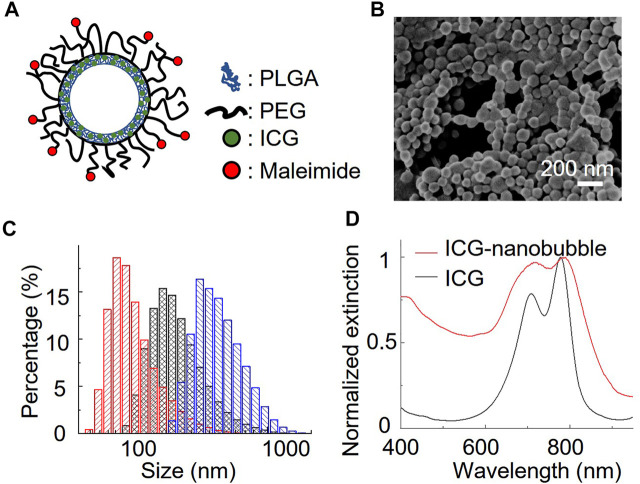
Characterization of ICG nanobubbles. **(A)** The structural illustration of an ICG nanobubble. **(B)** Environmental-SEM image of ICG nanobubbles (100 nm). **(C)** DLS measurement of ICG nanobubbles. The red, black and blue bar chart represent the size distribution of ICG-nanobubbles with the mean size of 129.3 ± 65.3 nm, 259.2 ± 137.4 nm, and 353.8 ± 153.1 nm. **(D)** The extinction spectrum of ICG and ICG nanobubbles (350 nm).

We chose a biodegradable poly (lactic-co-glycolic acid) (PLGA) to synthesize ICG-loaded nanobubbles. PLGA is an FDA-approved elastomeric copolymer. Compared to other conformations of ICG-encapsulated ultrasound contrast agents, such as ICG-conjugated lipid nanobubbles and ICG-doped mesoporous silica nanoparticles, PLGA shell accommodates a large number of dyes in their 3D polymer network. PLGA network can also prevent the ICG dyes from being exposed to the environment, resulting in unwanted optical property changes. The procedure to prepare ICG-PLGA microbubbles and nanobubbles has been well documented; thus, a straightforward candidate for us to study the effect of the gas bubble on photoacoustic characteristics. Here we chose the double emulsion method to prepare ICG-PLGA nanobubbles with a size range from 100 nm to 350 nm (Figure 1B; Supplementary Figure S1). We first emulsified ICG/PVA/water solution with PLGA/CH_2_Cl_2_ solution using a dip-in sonicator. To prevent the non-specific binding and add functional groups for antibody conjugation in later *in vivo* applications, we include 10 wt% of poly(lactide-co-glycolide)-b-poly(ethylene glycol) (PLGA-PEG), and 10 wt% of poly(lactide-co-glycolide)-b-poly(ethylene glycol)-maleimide (PLGA-PEG-MAL) copolymer in the PLGA solution. Once the first emulsion was formed, they were quickly transferred to PVA/water solution under sonication to form the second double emulsion. The emulsion solution was then added to the isopropanol/water solution to evaporate the dichloromethane under gentle magnetic stirring. The final ICG-PLGA nanoparticles were freeze-dried in a lyophilizer to remove the water and form nanobubbles. The size of the ICG-PLGA nanobubbles can be controlled by the combination of PVA concentrations and the power of sonification. Large-size nanobubbles required a reduced concentration of PVA and sonication power. To evaluate the effect of the gas core on its photoacoustic signal, we prepared three different sizes of ICG nanobubbles, measured the size distribution with DLS, and confirmed with environmental SEM. As shown in Figure 1C, the DLS measurement shows the average size of ICG nanobubbles is 129.3 ± 65.3 nm, 259.2 ± 137.4 nm, and 353.8 ± 153.1 nm, respectively. They are referred to as “100 nm”, “250 nm”, “350 nm” ICG nanobubbles in the following to improve the readability. We further test the stability of the ICG PLGA nanobubbles in physiologically relevant PBS solution by tracking their size for 48 h using DLS. The result (Supplementary Figure S2) shows no significant change in size distribution, indicating that the nanobubbles are stable for at least 48 h in PBS.

### 3.2 Photoacoustic/ultrasound signal characterization of ICG nanobubbles

We first test the photoacoustic signal stability of ICG PLGA nanobubbles. We conducted a photothermal stability test by recording the photoacoustic amplitudes of free ICG and ICG PLGA nanobubble solutions as functions of the laser pulses. We recorded 1,000 photoacoustic responses of the samples in a tube phantom with an average laser fluence of 10 mJ/cm^2^, which is larger than the damage threshold of the free ICG solution. As shown in Supplementary Figure S3, the photoacoustic amplitude of the free ICG solution decays over time because of the photothermal damage. In contrast, the photoacoustic amplitude of ICG PLGA nanobubble remains no change for at least 1,000 laser pulses.

**FIGURE 2:**
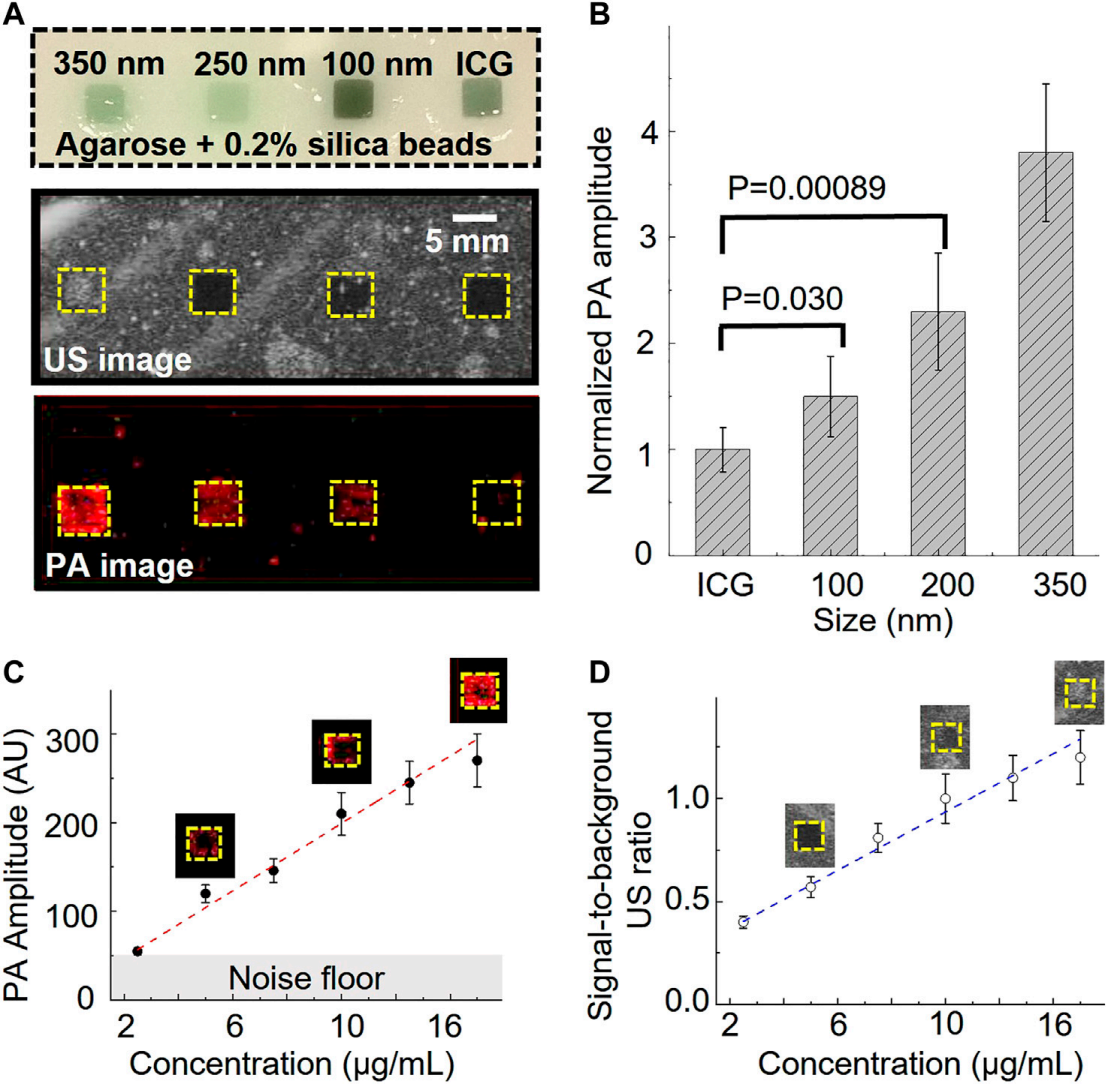
Photoacoustic/ultrasound signal characterization of ICG nanobubbles. **(A)** Ultrasound/photoacoustic image of 350 nm ICG nanobubbles, 250 nm ICG nanobubbles, 100 nm ICG nanobubbles and ICG solution. The four solutions were filled into the four inclusions of the agarose phantom, indicated by the picture of agarose phantom on the top. All four solutions match with the same optical density at 780 nm. **(B)** Quantitative comparison of photoacoustic signal of different solutions (ICG, 100 nm ICG nanobubbles, 250 nm ICG nanobubbles, 350 nm ICG nanobubbles). Bar chart showing sample means (n = 10) with standard-deviation error bars. **(C)** PA signal as a function of ICG nanobubble concentration. 350 nm ICG nanobubbles were used and the curve shows PA signal is propositional to the bubble concentration. Bar chart showing sample means (n = 10) with standard-deviation error bars. **(D)** Signal-to-background ratio of ultrasound signal as the function of ICG nanobubble concentration. 350 nm ICG nanobubbles were used and the curve shows a linear relationship. Bar chart showing sample means (n = 10) with standard-deviation error bars.

Further, we prepared an agarose phantom with four squared gelatin inclusions to characterize the ultrasound and photoacoustic performance. The agarose phantom contained 0.5 wt% of silica beads (40 µm) to mimic tissue ultrasound scattering (Cook et al., 2011). The phantom contained four gelatin (5 vol%) inclusions. The first three inclusions included 50 vol% of 350 nm, 250 nm, and 100 nm nanobubble solutions, respectively (Figure 2A). The nanobubble concentration was matched based on the optical density (OD) of the ICG optical absorption at 780 nm. The fourth gelatin inclusion (50 vol%) contained OD-matched free ICG aqueous solution and was used as a control (Figure 2A). The images were recorded with a 21 MHz ultrasonic probe and laser fluence at 5.5 mJ/cm^2^ at 780 nm. As shown in Figure 2A, the inclusions with 350 nm nanobubbles show significant ultrasound contrast enhancement but not the rest of the inclusions. Interestingly, each nanobubble inclusion showed a significant photoacoustic signal enhancement compared to that with the same concentration of free ICG dye.

To better understand this enhancement effect, we quantified the photoacoustic signal by calculating the averaged photoacoustic signals of each pixel within the inclusions and then normalized the signal of each nanobubble with the averaged signal of free ICG (Figure 2B). The signal enhancement is strongly affected by the nanobubble sizes (Figure 2). The result shows a 1.5 ± 0.28 times enhancement for 100 nm nanobubbles, 2.3 ± 0.55 times enhancement for 250 nm nanobubbles, and 3.8 ± 0.65 times enhancement for 350 nm nanobubbles.

Based on the general photoacoustic theory, the photoacoustic signal intensity should be mainly determined by the optical absorption of the samples if laser fluence and thermal properties (such as Grüneisen parameter) of the samples are the same (Xu and Wang, 2006). Because optical scattering increase as nanobubble size increases, if taking optical scattering into consideration, we expect the free ICG solution will produce largest photoacoustic signal and the 350 nm nanobubbles generate the weakest photoacoustic signal, but the results show the opposite.

It is known that photoacoustic signals can change when nanoparticles are closely packed (Chen et al., 2017). To rule out the possibility that the photoacoustic signal enhancement is caused by the nanobubble aggregation, we conducted separate phantom imaging to study photoacoustic signals as a function of nanoparticle concentrations. If the enhancement is caused by the aggregation of the nanobubbles, we expect the photoacoustic signal will increase non-linearly as the concentration increases. In this study, 350 nm nanobubbles, which produce the strongest signal among our samples (Figures 2A, B), were used for investigating the potential non-linear effect. The same imaging setup and phantom were used for this study. The result shows that the photoacoustic and ultrasound signal have positive linear correlations with the concentration of nanobubbles (Figure 2C, D). The result suggests that nanobubbles are well dispersed in the gelatin matrix, and the photoacoustic signal enhancement is not due to the nanoparticle aggregation.

Our prior research shows that in nanoparticle samples, thermal property changes of nanoparticle coating (or surrounding), and the aggregation of nanoparticles (or optical absorbers) can alter the photoacoustic signal (Chen et al., 2011; Chen et al., 2017). The densely packed ICG in the shell and the change of the surrounding thermal properties can be the attributions to the photoacoustic signal difference between free ICG solution and the ICG nanobubbles. However, these effects cannot explain the photoacoustic difference between ICG nanobubbles with various sizes since same ratio of ICG/PLGA were used in preparing the different size nanobubbles, meaning similar separation between dyes. Thus, there is an additional factor that is size dependent causing the photoacoustic enhancement.

### 3.3 Theoretical calculations of photoacoustic response of ICG nanobubbles

We conducted the theoretical analysis to investigate the effect of the gas core on the photoacoustic response in ICG nanobubbles. The photoacoustic signal of a nanoparticle-coated microbubble has been studied (Firouzi et al., 2013; Dixon et al., 2015; Kuriakose and Borden, 2021). This prior study shows that the photoacoustic signal generated in optical absorbing nanoparticles can interact and cause the oscillation of the microbubble. The overall photoacoustic signal of a microbubble is the combination of nanoparticle photoacoustic signal and the microbubble oscillation. From the nanobubble’s structure and based on our observations of the size-dependent photoacoustic enhancement, we hypothesized that the photoacoustic signal generation may be strongly affected by the oscillation of the gas core as well. To test the hypothesis, we conducted theoretical calculations of ICG nanobubble photoacoustic response (see “Method”). Our calculation focused on investigating the size effect of the nano-size air bubble on the photoacoustic signal. We assumed that photoacoustic signals generated from ICG dyes in each nanobubble are the same and calculated how the air bubble of various sizes interact with this photoacoustic signal.

**FIGURE 3:**
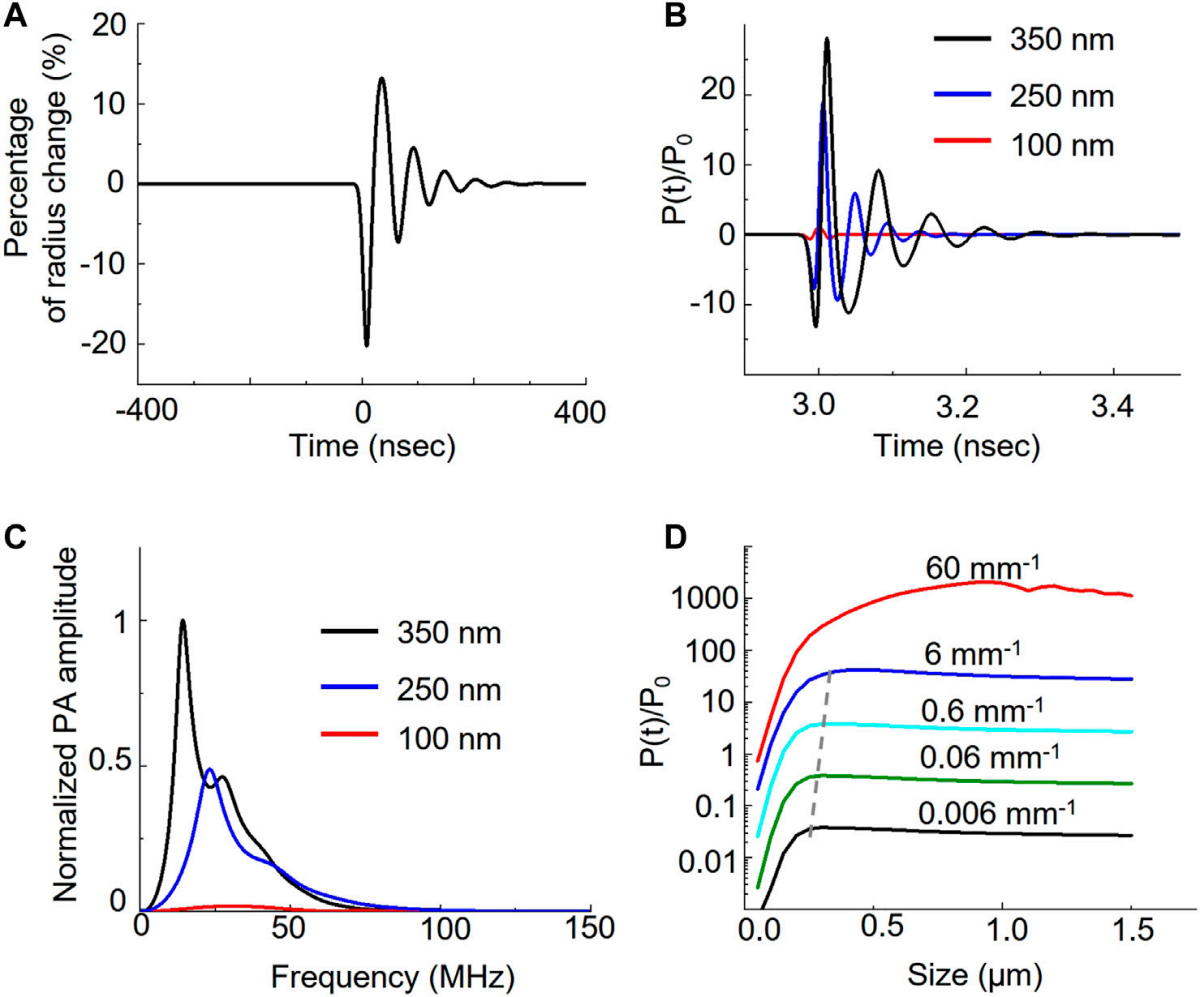
Theoretical analysis of nanobubble induced photoacoustic enhancement. Laser irradiation induces photoacoustic pressure on the ICG shell of the nanobubble. The pressure further induces the oscillation of nanobubble. **(A)** The bubble radius oscillates over time when interacting with photoacoustic signal of ICG dyes. **(B)** Different bubble size results in the different pressure distribution. The photoacoustic pressure, P(t), is normalized to ambient pressure, P_0_. **(C)** Frequency response of the photoacoustic pressure in different sizes. **(D)** Comparison of the photoacoustic pressure as a function of nanobubble sizes in various optical absorption of ICG.

To investigate how the nanobubble oscillation affects the overall photoacoustic signal, we used the Rayleigh-Plesset model to solve the photoacoustic pressure distribution over time (Marmottant et al., 2005; Firouzi et al., 2013). Figure 3A shows that initial photoacoustic pressure from ICG can cause nanobubble oscillation. The ICG photoacoustic pressure first compresses the nanobubble by 20% in radius and then causes the oscillation. The oscillation generates a photoacoustic signal in addition to the original ICG signal. Because sizes determine the resonance of the oscillation, it affects the photoacoustic amplitude and the corresponding frequency response. Figure 3B shows that the oscillations induce 18-fold and 28-fold signals in 250 nm and 350 nm nanobubbles compared to the 100 nm nanobubbles, while the experiments show only 1.5-fold and 2.5-fold enhancement. The discrepancy between experiments and calculations can be partially attributed to the different medium and confinement assumptions in the calculation. The medium is water in our calculations, and it is gelatin in our phantom experiments. We assumed thermal confinement in our calculation, which over-estimate the photoacoustic signal. Further, dense packing of ICG dye in the shell can contribute to the enhancement as well. It is known that when the distance between the optical absorbers is less than the thermal diffusion length, the photoacoustic signal can be enhanced due to build-up temperature from overlapping the thermal profile with adjacent optical absorbers (Chen et al., 2017). This effect is not taken into account in the calculation because the goal of the calculation is to compare the size effect of the gas core. In Figure 3C, we show that the different sizes cause different frequency responses. By analyzing the frequency response of the photoacoustic signal generated by nanobubble oscillation, we found that the peak frequency shift from 14.5 MHz, 23 MHz, to 30.5 MHz when the size of nanobubble reduces from 350 nm, 250 nm, to 100 nm, respectively.

We also analyzed the photoacoustic signal as a function of nanobubble sizes as well as the ICG absorption. We calculated the effect of ICG optical absorption from 0.006 mm^−1^ to 60 mm^−1^ with a 10-fold interval on the photoacoustic signal with nanobubble sizes up to 1.5 μm. Our results show that the photoacoustic signal increases linearly with the absorption coefficient below 6 mm^−1^. When increasing the optical absorption to 60 mm^−1^, the photoacoustic signal, especially from large nanobubbles, deviates from the linear relation. We also observed the size of the peak photoacoustic signal is not a constant but a function of the absorption coefficient. The peak size shifts from 300 nm to 450 nm linearly when the optical absorption increases from affected 0.006 mm^−1^ to 6 mm^−1^; however, it shifts drastically to ∼1 µm in 60 mm^−1^ of the optical absorption. We further calculated the normalized photoacoustic signal as a function of nanobubble size in various laser pulse widths. The result (Supplementary Figure S4) shows that as laser pulse width increases, the photoacoustic signal enhancement increases, but the enhancement peak shifts to a larger nanobubble size.

### 3.4 Molecular specificity and cytotoxicity of GRPR-targeted ICG nanobubbles

**FIGURE 4:**
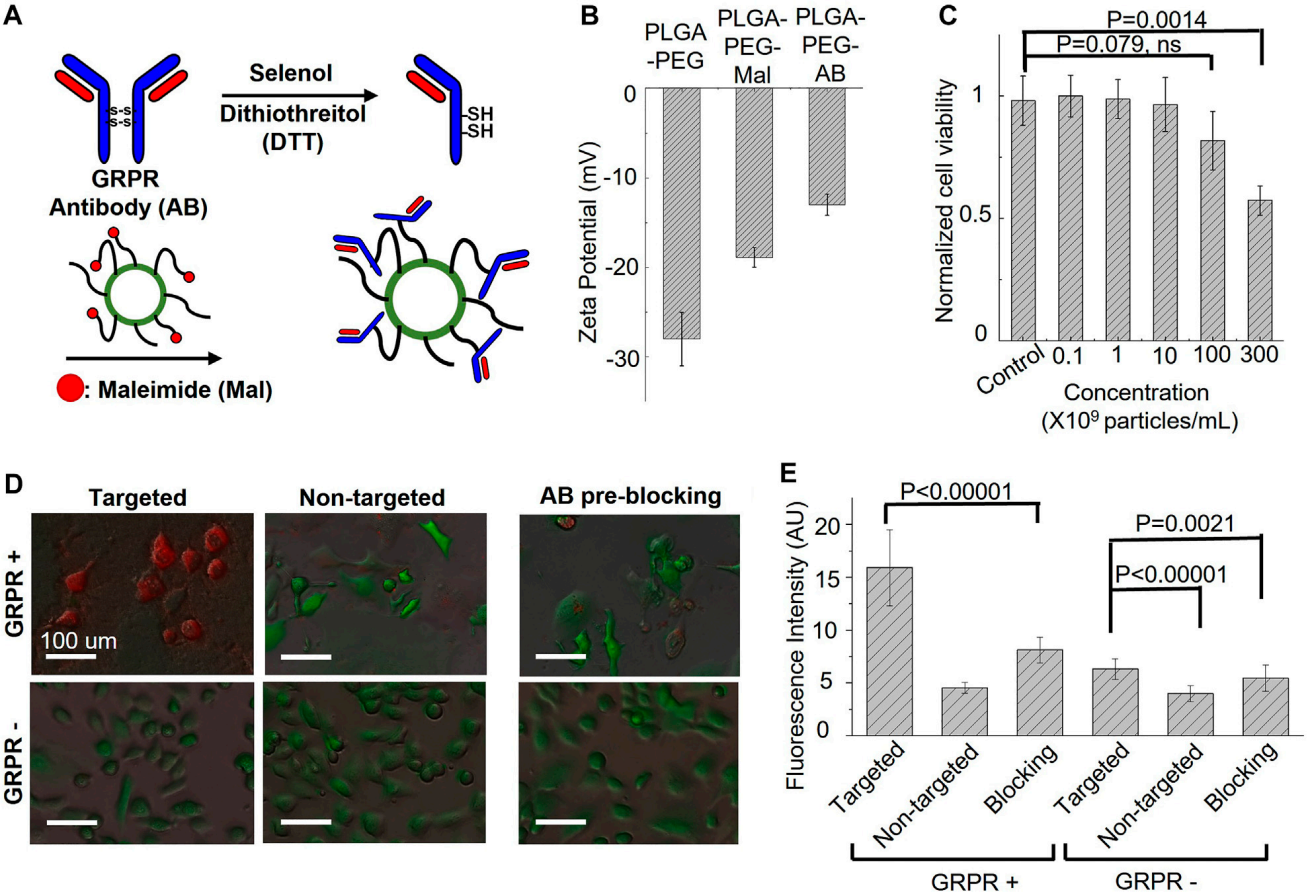
Targeting specificity and cytotoxicity of GRPR-targeted ICG nanobubbles. **(A)** The synthesis steps for anti-GRPR antibodies conjugation with an ICG nanobubble. **(B)** Zeta-potential of ICG nanobubbles (PLGA-PEG) with different fictionization (with and without maleimide groups and conjugated with anti-GRPR antibody). Bar chart showing sample means (n = 3) with standard-deviation error bars. **(C)** Cell viability of GRPR+ prostate cancer cells as the function of the concentration of ICG nanobubble incubated with the cells. Bar chart showing sample means (n = 5) with standard-deviation error bars. **(D)** The fusion of bright-field and fluorescent images of ICG nanobubbles incubated with PC3 (GRPR+) and DU145 (GRPR-) cells. All cells were genetically encoded with green fluorescent protein (Green color). Red color represents ICG fluorescence signal. **(E)** Fluorescent intensities of ICG from PC3 (GRPR+) cells or DU145 (GRPR–) cells incubated either with targeted or non-targeted IGC nanobubbles. Higher intensities reflect higher targeting efficiency. Bar chart showing sample means (n = 5) with standard-deviation error bars.

Gastrin-releasing peptide receptor (GRPR) is our target of interest for *in vivo* molecular imaging, as it is a biomarker overexpressed in many cancers, including prostate, breast, colon, and lung cancers. To develop GRPR-targeted ICG nanobubbles, we conjugated GRPR antibodies with ICG nanobubbles. Specifically, we reduced the disulfide bonds of anti-GRPR antibodies with DTT as a reducing agent and selenol as a promoter, then conjugated thiol groups of split antibodies with the maleimide groups on PLGA nanobubbles. To confirm each surface functionalization, we measured the zeta potential of the ICG-PLGA-PEG nanobubbles with and without maleimide groups, and the nanobubbles conjugated with anti-GRPR antibody (anti-GRPR-ICG nanobubbles). As presented in Figure 4B, the ICG-PLGA-PEG nanobubble shows a significant negative zeta potential (−28 ± 3 mV), coming from the negatively charged acidic group in PLGA. ICG-PLGA-PEG nanobubble is used as a control sample to determine the change of each functionalization step on the nanobubble surface. To produce nanobubble with maleimide groups, 20 wt% of PLGA-PEG is replaced with PLGA-PEG-Maleimide during synthesis. The zeta potential of nanobubbles with maleimide groups increases to −18.9 ± 1.1 mV because of the positive charge of maleimide groups. After antibody conjugation, the zeta potentials of the nanobubbles further increase to −13.0 ± 1.2 mV due to the slightly positively charged amino acids.

Before the *in vitro* affinity study, cytotoxicity of the GRPR-targeted nanobubbles on GRPR+ prostate cancer cells, PC3, was evaluated using Presto Blue cell viability assay. The result shows no obvious reduction in cell viability when cells were incubated with up to 1 × 10^10^ nanobubbles/mL for 24 h. While we observed a reduction of averaged viability drop to 0.82 ± 0.12 with 1 × 10^11^ nanobubbles/mL, the *p*-value is 0.079 (N = 5), indicating the insignificant difference with control. The nanobubble started to show significant toxicity at the concentration of 3 × 10^11^ nanobubbles/mL, which reduced the averaged viability to 0.57 ± 0.06 (Figure 4C, *p* = 0.0014, N = 5).

To test whether the conjugated antibodies retain their affinity to GRPR, both GRPR+ (PC3) and GRPR− (DU145) cell lines were incubated with either targeted (GRPR) or non-targeted (PEG) nanobubbles (1×10^8^ nanobubbles for 1 × 10^5^ cells) for 2 hours. GRPR+ cells show higher ICG fluorescence signals (red color) when incubated with GRPR-targeted nanobubbles compared to non-targeted nanobubbles (Figure 4D). As expected, the negative control cell line shows negligible fluorescence signals when incubated with either targeted or non-targeted nanobubbles (Figure 4D). We further performed a blocking study where GRPR + cells were pre-incubated with anti-GRPR antibody (100 ng) in excess for 30 min prior to incubation with GRPR-targeted saline nanobubbles. The pre-incubation with anti-GRPR antibody significantly decreases the binding of the nanobubbles, as shown by the lower ICG fluorescence signals compared to that without blocking (Figure 4E). We quantitatively analyzed the fluorescence signal on the fluorescence images. The ICG fluorescence signal of the GRPR+ cells is 3.5-fold (*p* < 0.00001, N = 36) higher when incubated with GRPR-targeted nanobubbles compared to non-targeted nanobubbles. The signal is twice (*p* < 0.00001, N = 36) higher when compared with the signal of the GRPR + cells, which are first blocked by anti-GRPR antibodies for 30 min before incubating with GRPR-targeted nanobubbles. To compare with GRPR− cells, the signal of GRPR+ with GRPR-targeted nanobubbles is 2.5-fold (*p* < 0.00001, N = 36), 4-fold (*p* < 0.00001, N = 36), and 2.9-fold (*p* < 0.00001, N = 36) higher when comparing with the ICG signal of the three cases of GRPR− cells (−/+, −/−, pre-blocking −/+). The result confirms the GRPR-targeted nanobubble is molecularly specific to GRPRs.

### 3.5 *In vivo* dual photoacoustic/ultrasound imaging of prostate cancer using ICG nanobubbles

**FIGURE 5:**
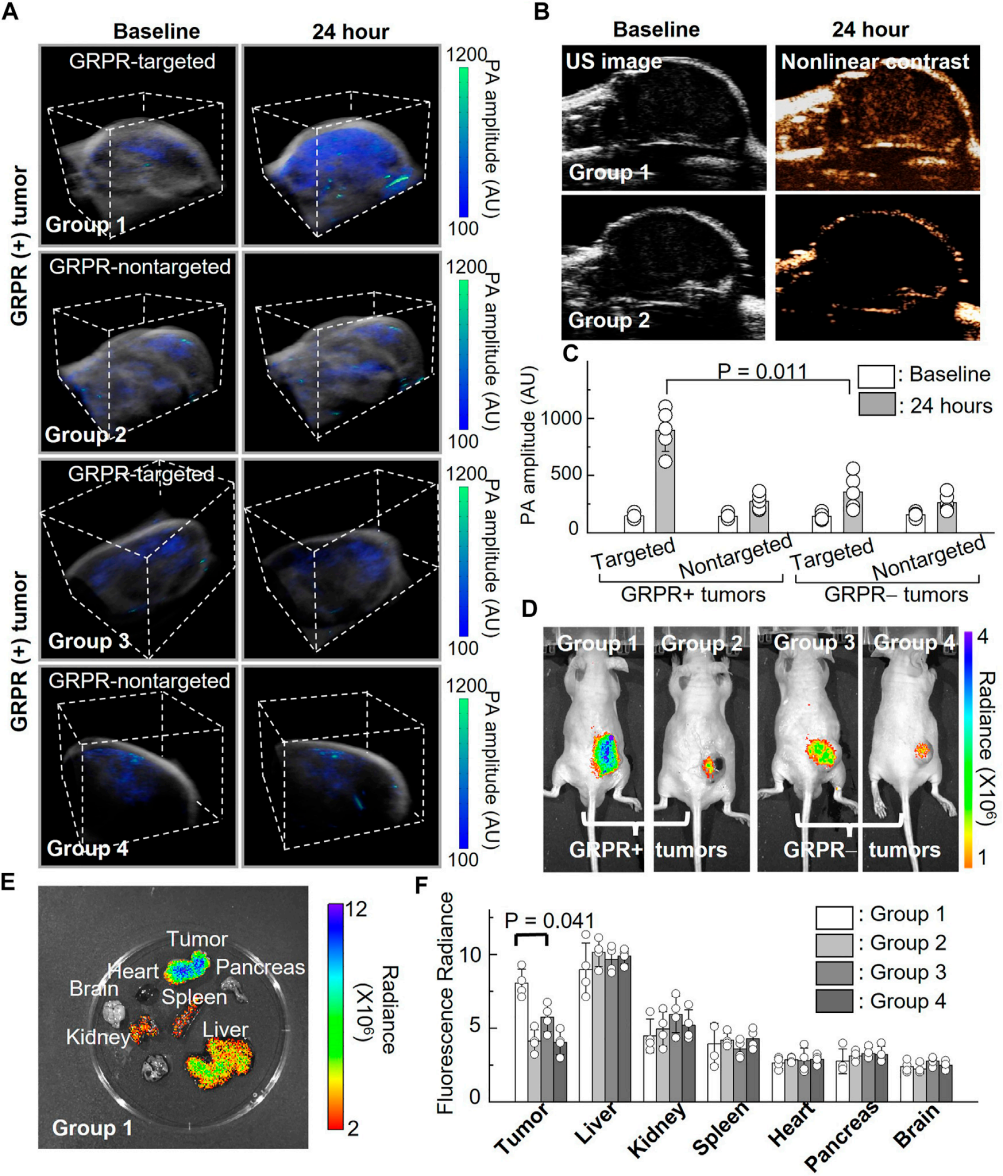
*In vivo* imaging of ICG nanobubbles in a murine model of prostate cancer. **(A)** Dual modal 3D photoacoustic/ultrasound images of GRPR+ and GRPR-tumor-bearing mice injected with non-targeted or targeted ICG nanobubbles (Groups 1–4). The scanning volume is 23 mm (x) × 19 mm (y) × 16 mm (z). **(B)** 2D ultrasound image and non-linear contrast ultrasound image of GRPR+ tumor-bearing mice with non-targeted or targeted ICG nanobubbles (Groups 1–2). **(C)** Photoacoustic signal of GRPR+ and GRPR- tumor-bearing mice injected with non-targeted or targeted ICG nanobubbles (Groups 1–4). Bar chart showing sample means (n = 5) with standard-deviation error bars. **(D)** Fluorescence images of tumor-bearing mice in Groups 1 to 4. **(E)** A representative fluorescence image of harvested kidney, brain, tumor, heart, spleen, pancreas and liver from the tumor-bearing mouse in Group 1. **(F)** Fluorescence intensities from the main organs and tumors from the mice in Groups 1–4. Bar chart showing sample means (n = 4) with standard-deviation error bars.

To investigate the feasibility of GRPR-targeted ICG nanobubbles as molecular imaging agents, we first tested the GRPR targeting ability of nanobubbles in mouse models of prostate cancer. We implanted 2 × 10^6^ GRPR+ (PC3, group 1 and group 2) and GRPR− (DU145, group 3 and group 4) prostate cancer cells subcutaneously to the right flanks of male nu/nu mice (N = 5 for each group). Before the injection of nanobubbles, we scanned every tumor of the mice using a photoacoustic/ultrasound imaging system to generate 3D baseline images. We injected the mouse intravenously with 200 µL of active nanobubbles (GRPR targeted ICG nanobubbles) to group 1 and group 3, and control (PEG-ICG nanobubble) nanobubbles to group 2 and group 4 at a concentration of 10 mg/mL. 24 h after injection, photoacoustic/ultrasound imaging of all mice was performed to monitor nanobubble distribution in tumors. A representative photoacoustic image of tumors from each group is shown in Figure 5A, showings visibly higher photoacoustic signals from the GRPR+ tumor with GRPR-targeted nanobubbles (group 1), compared to tumors in groups 2–4, indicating the *in vivo* GRPR-specificity of our targeted nanobubbles. Representative ultrasound and non-linear ultrasound images of tumors from groups 1 and 2 were shown in Figure 5B, showing higher ultrasound signals from targeted nanobubbles than non-targeted nanobubbles. Quantitative analyses (Figure 5C) of the photoacoustic signals of the tumors also show at least 3.0 ± 1.4-fold (N = 5) higher signal in group 1 than in groups 2–4.

Immediately after photoacoustic/ultrasound imaging, whole-body fluorescence imaging (IVIS) was performed to confirm the successful delivery and targeting of the ICG nanobubbles. Figure 5D shows a representative ICG fluorescence image of each group; the higher ICG fluorescence signals in the tumors of group 1 compared to groups 2–4 further confirmed the specificity of the targeted nanobubbles to GRPR+ tumors, which corroborated with our photoacoustic/ultrasound imaging results (Figure 5D). After *in vivo* imaging, we sacrificed the mice and excised the key organs (heart, liver, kidney, spleen, pancreas, and brain) and tumors to assess the bio-distribution of the ICG nanobubbles (Figures 5E, F). Figure 5F shows that the fluorescence signals in the tumors of group 1 are significantly higher than the rest of the groups (2.2 ± 0.4-fold, 1.4 ± 0.3-fold, and 2.0 ± 0.2-fold higher than groups 2, 3, and 4, respectively). Furthermore, fluorescence quantification shows that in addition to tumor uptake, both targeted and non-targeted ICG nanobubbles mainly accumulate in the liver and moderately accumulate in the kidney and spleen, while negligible signals were detected in the other organs due to the elimination by the reticuloendothelial system.

## 4 Discussion

Photoacoustic imaging is a low-cost, portable, and non-ionizing imaging technique that is emerging in many clinical applications. It can be used with molecular imaging agents to offer additional molecular and cellular information on diseases. This combination has been demonstrated extensively in preclinical research with many successes. Much current effort has been invested in translating the exciting preclinical results to clinics. One key element of a successful clinical translation is an imaging agent that can be safely used in humans. The development of imaging agents focuses on constructing imaging agents from translatable materials to minimize the risk of unexpected toxicity during translation. However, biocompatible materials may not provide the best photoacoustic response, thus significantly limiting the material choice when producing imaging agents with high photoacoustic intensity. The low photoacoustic signal requires relatively higher molecular agents and reduces the imaging sensitivity.

In this study, we developed ICG PLGA nanobubbles. The main constitutional materials, ICG and PLGA, are approved by FDA and have been widely used in many medical applications. Many research efforts have been devoted to synthesizing ICG nanobubbles as translatable photoacoustic agents. We discovered the photoacoustic response of the ICG dyes in nanobubbles could be improved up to almost four-fold compared with the same concentration of the free dyes. Our theoretical calculation demonstrated that ICG photoacoustic signal could vibrate the nanobubble core and the signal can be enhanced through the resonance of the gas cavity, and the signal enhancement is nanobubble size dependent. We further showed that our ICG nanobubbles can also produce sufficient ultrasound contrast for dual photoacoustic/ultrasound imaging. We then developed a chemical approach to conjugate the nanobubbles with molecularly targeted ligands and anti-GRPR antibodies to produce GRPR-targeted nanobubbles. With these signal-enhanced molecular targeted nanobubbles, we demonstrated an *in vivo* dual-photoacoustic/ultrasound molecular imaging of GRPR in a mouse model of prostate cancer.

We expect these findings will point out an alternative route to circumvent the limitations of the materials and accelerate the development of translatable photoacoustic/ultrasound molecular imaging for other cell surface targets and various disease states. Although we demonstrated the nanobubbles with PLGA, the same effect should be valid for other dye-doped nano- or microbubbles.

## Data Availability

The original contributions presented in the study are included in the article/Supplementary Material, further inquiries can be directed to the corresponding author.
