# Supplementary material for: Photoacoustic signal enhancement in dual-contrast gastrin-releasing peptide receptor-targeted nanobubbles
Source: Front Bioeng Biotechnol. 2023 Jan 17;11:1102651. doi: 10.3389/fbioe.2023.1102651 (PMC9887164; doi:10.3389/fbioe.2023.1102651)
Supplement: Supplementary file 1 [file DataSheet1.PDF]

Supplementary Information for "Photoacoustic signal enhancement in dual-contrast gastrin-releasing peptide receptor-targeted nanobubbles"

Shensheng Zhao, Leanne Lee, Yang Zhao, Nu-Chu Liang, Yun-Sheng Chen

Supplementary Tables

Table 1: simulation parameters

Region	Parameter	Symbol	Unit	Value
Gas	Polytropic constant	κ	-	1.4
Liquid	Density	ρ_L	1000	kg/m^3
	Viscosity	μ_L	$8.9\text{x}10^{-4}$	Pa·s
	Surface tension	σ	0.02	N/m
	Ambient pressure	P_0	0.1	MPa
Dye	Optical absorption coefficient	μ_a	6	mm^{-1}
	Gruneisen parameter	Γ	0.11	-
	Sound speed	c	1540	m/s

Supplementary Figures

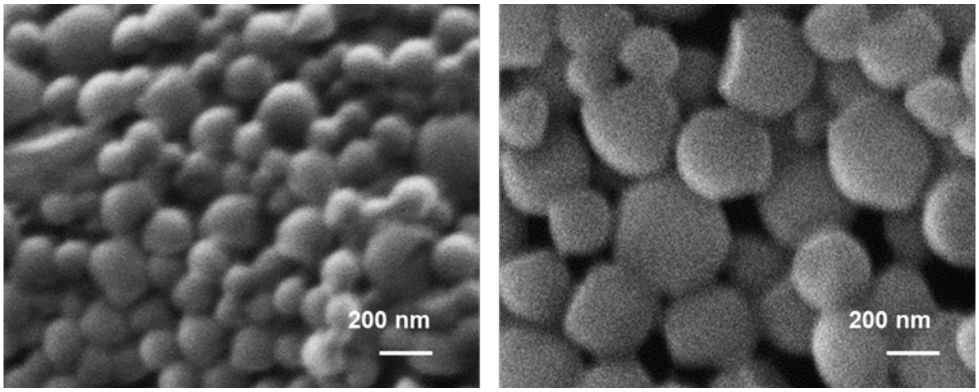

Supplementary Figure S1| Environmental scanning electron microscopy images of ICG nanobubbles. The images show the nanobubble with an average size of (a) 259.2 ± 137.4 nm, and (b) 353.8 ± 153.1 nm.

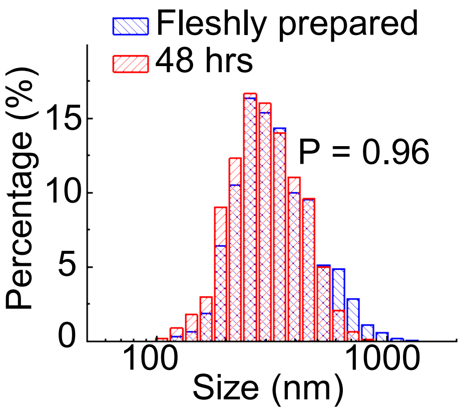

Supplementary Figure S2| Stability of ICG nanobubbles analyzed by size distribution using DLS measurement. The blue chart represents the size distribution of freshly prepared ICG nanobubbles/PBS solution. The mean size of the freshly prepared nanobubble is 353.8 ± 153.1 nm. The red chart represents the size distribution of the same ICG nanobubbles in PBS after 48 hours of preparation (red). The mean size of the nanobubble is 322.9 ± 115.7 nm. The P value (two-tail t-test) of the two size distributions is 0.96 indicating there is no significant difference ($P > 0.5$) between the two samples.

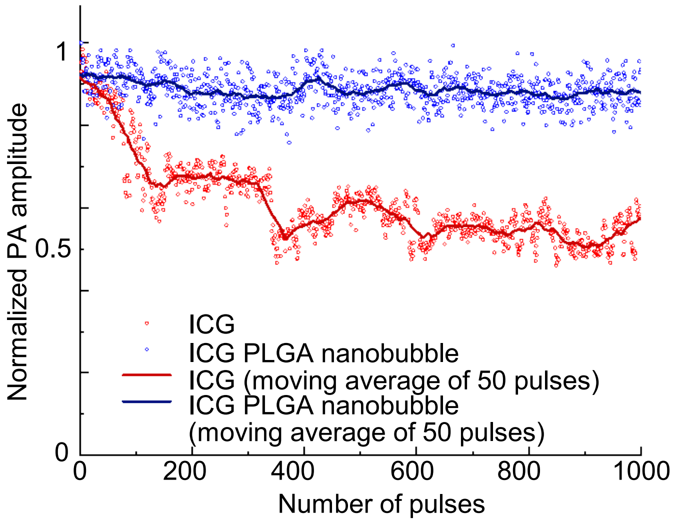

Supplementary Figure S3| Photoacoustic signal stability test of free ICG solution and ICG PLGA nanobubble solution, showing ICG PLGA nanobubbles have better photothermal stability under pulsed laser exposure. Free ICG and ICG PLGA nanobubble solutions with matched OD at 780 nm are illuminated with 1000 laser pulses (average laser fluece is 10 mJ/cm^2). The photoacoustic response of each pulse was recorded using a linear array transducer (21 MHz). The solid line is the moving average of 50 pulses.

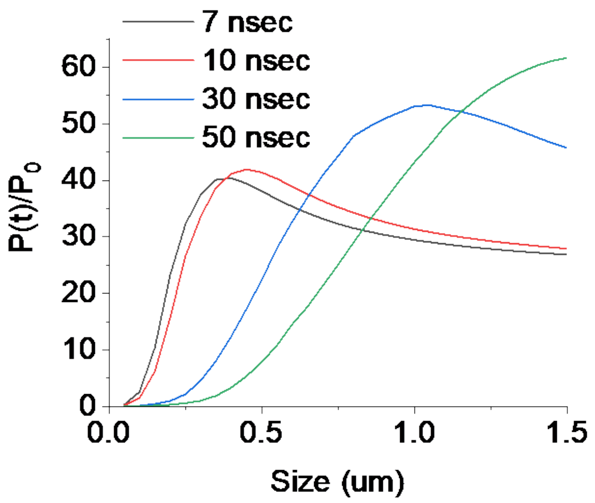

Supplementary Figure S4| Comparison of the normalized photoacoustic pressure as a function of nanobubble sizes in various laser pulse widths in 7, 10, 30, and 50 nanoseconds. The result shows that while the large laser pulse produces increasing enhancement, the long laser pulse width shifts the resonance of the nanobubble to a large size.
